# Book review: The Epigenetics Revolution

**DOI:** 10.3389/fgene.2015.00250

**Published:** 2015-08-13

**Authors:** Cieron Roe, Lucy May

**Affiliations:** Brighton and Sussex Medical SchoolBrighton, UK

**Keywords:** genetics, epigenetics, inheritance, cancer, therapeutics, books, review

Epigenetics is a rapidly evolving science that is often only described in scientific literature or textbooks. In “The Epigenetics Revolution”, Nessa Carey eloquently bridges the spheres of academia and scientific journalism (Carey, 2012). The phrasing “revolution” is a dramatic use of English that effectively portrays a momentous shift in biological thinking. For the novice, it is a good introduction to epigenetics whilst it provides professional readers with a concise summary of historically significant experiments and translational context.

The text is divided into an introduction and 16 chapters which handle various concepts pertaining to epigenetic theory, key experimental studies and the potential for future therapeutic application. Carey starts with some historical context; she illustrates the successes and limitations of the human genome project and quotes some of the arguably overdramatic claims made by various organizations of the time. The first two chapters approach the issue of cellular differentiation by explaining the work of John Gurdon and the concept of a genetic “switch” rather than a loss of unneeded material. The analogy that DNA is a script to be interpreted is a particularly articulate explanation for novice readers. From this, Carey explains the epigenetic underpinnings of pluripotency and explores the potential application of stem cell therapies in regenerative medicine. Chapters 3 and 4 give an overview of genetic and epigenetic molecular biology, including the genetic code, DNA replication, transcription, translation, chromatin, DNA methylation, and histone modifications. These biological concepts are supplemented with an explanation of twin studies to illustrate the profound effect of environmental experience on genetically identical individuals. She makes reference to specific biological signals that induce epigenetic modification e.g. the hormone receptor. In the absence of somatic mutation, Carey also explores how epigenetic modifications can induce cellular abnormalities. Overall, this section provides an excellent narrative that uses famous scientific work to illustrate basic epigenetic concepts which is perfectly suited to novice readers or undergraduate students.

Chapter 6 focuses on the heritability of epigenetic modifications. Environmental events such as the Dutch famine have clear effects on the health outcomes of offspring. Carey explains that this only occurs in susceptible regions of the genome and that epigenetic reprogramming during early embryogenesis circumvents the passage of many undesirable or dangerous modifications. Generation X is a very interesting chapter that explains the history of sex determination; an epigenetic process that depends upon tight regulation of X chromosome genes, termed “X chromosome dosage compensation.” The last quarter of the book focuses on epigenetic pathology. Carey illustrates this section with two very different disease states; psychiatric illness and cancer. This is purposefully designed to portray the universal influence of epigenetics. Carey makes an intelligent case for the role of childhood experience in shaping the epigenetic landscape; specifically, the association between abuse and the development of mental illness. Another interesting section explores the current contention surrounding neuro-epigenetics. This implies that epigenetic theory isn't sacrosanct and provokes a good debate for the reader. Disappointingly, neurodegeneration is only briefly mentioned. On the contrary, the concept of “epigenetic” aging is thorough and chapter 13 eloquently explains the global decrease in methylation and changes to the telomere system that are associated with the development of cancer. The final chapter elegantly draws together the biological principles, historical events, and translational examples that were given throughout the book. Carey concludes by exploring the potential for future drug discovery and the limitations and dangers that such agents may present. There is a description of recent research that is beginning to answer many elusive questions, such as the mechanisms underpinning the circadian rhythm. This section builds upon basic concepts with more advanced theory which might become challenging for some inexperienced readers. With that said, it is brilliantly written and provides a comprehensive review of many historically significant feats of epigenetic biology.

“The Epigenetics Revolution” is a concise text that is accessible to a wide audience. However, such a compromise presents drawbacks; for the absolute beginner, gene nomenclature and complex scientific concepts may be confusing at times. If one is familiar with the field, many of the examples are unoriginal e.g. the agouti mouse experiments, the classic Dutch famine and its correlation with obesity, the royal jelly etc. Surprisingly, there is no mention of the early work by nobel laureates Barbara McClintock or Paul Berg. Despite these relatively minor criticisms, “The Epigenetics Revolution” is an excellent scientific biography that charts the past, present, and optimistic future of the field. It is especially suitable for informed non-specialists and students of biological sciences. Carey enthusiastically portrays a revolutionary system that could unlock the answers to many of nature's best kept secrets.

## Conflict of interest statement

The authors declare that the research was conducted in the absence of any commercial or financial relationships that could be construed as a potential conflict of interest.

## References

[B1] Carey N. (2012). The Epigenetics Revolution: How Modern Biology is Rewriting Our Understanding of Genetics, Disease and Inheritance. London: Icon Books Ltd.

